# Lung Sounds in Children before and after Respiratory Physical Therapy for Right Middle Lobe Atelectasis

**DOI:** 10.1371/journal.pone.0162538

**Published:** 2016-09-09

**Authors:** Satoshi Adachi, Hiroshi Nakano, Hiroshi Odajima, Chikako Motomura, Yukiko Yoshioka

**Affiliations:** 1 Department of Rehabilitation, National Hospital Organization, Fukuoka National Hospital, Fukuoka, Japan; 2 Department of Pulmonology, National Hospital Organization, Fukuoka National Hospital, Fukuoka, Japan; 3 Department of Pediatrics, National Hospital Organization, Fukuoka National Hospital, Fukuoka, Japan; Telethon Institute for Child Health Research, AUSTRALIA

## Abstract

**Background:**

Chest auscultation is commonly performed during respiratory physical therapy (RPT). However, the changes in breath sounds in children with atelectasis have not been previously reported. The aim of this study was to clarify the characteristics of breath sounds in children with atelectasis using acoustic measurements.

**Method:**

The subjects of this study were 13 children with right middle lobe atelectasis (3–7 years) and 14 healthy children (3–7 years). Lung sounds at the bilateral fifth intercostal spaces on the midclavicular line were recorded. The right-to-left ratio (R/L ratio) and the expiration to inspiration ratio (E/I ratio) of the breath sound sound pressure were calculated separately for three octave bands (100–200 Hz, 200–400 Hz, and 400–800 Hz). These data were then compared between the atelectasis and control groups. In addition, the same measurements were repeated after treatment, including RPT, in the atelectasis group.

**Result:**

Before treatment, the inspiratory R/L ratios for all the frequency bands were significantly lower in the atelectasis group than in the control group, and the E/I ratios for all the frequency bands were significantly higher in the atelectasis group than in the control group. After treatment, the inspiratory R/L ratios of the atelectasis group did not increase significantly, but the E/I ratios decreased for all the frequency bands and became similar to those of the control group.

**Conclusion:**

Breath sound attenuation in the atelectatic area remained unchanged even after radiographical resolution, suggesting a continued decrease in local ventilation. On the other hand, the elevated E/I ratio for the atelectatic area was normalized after treatment. Therefore, the differences between inspiratory and expiration sound intensities may be an important marker of atelectatic improvement in children.

## Introduction

Atelectasis is defined as a complete or partial collapse of a lung or lobe of a lung. It is not a rare complication during the course of bronchial asthma attacks or pneumonia in children [1] and occurs most frequently in the right middle lobe [2,3]. Although chest radiography is necessary for the diagnosis and follow up of atelectasis, in daily practice, chest auscultation provides clinically useful information that may reflect the effect of respiratory physical therapy (RPT) [4].

Atelectasis is accompanied by a decrease in local ventilation and structural changes of the pulmonary tissue in the affected area, which can change the intensity or spectra of breath sounds. An experimental study by Ploysongsang and colleagues demonstrated that crackles occur during the re-expansion of absorption atelectasis in the dependent lung [5]. In our clinical experience, there are often discrepancies between auscultation findings, such as crackles, and the clinical course of atelectasis. Therefore, the course of atelectasis is difficult to evaluate based on the status of crackles alone.

On the other hand, changes in the intensity and spectra of breath sounds have not been previously evaluated in subjects with atelectasis. Recently, the exact evaluation of lung sounds has become possible using computerized respiratory sound analysis [4,6], providing information that could be useful for improving auscultation skills.

The aim of this study was to clarify the characteristics of breath sounds and the changes in breath sounds before and after treatment including RPT, in children with atelectasis using computerized acoustic measurements. To our knowledge, this is the first reported study to assess breath sounds in subjects with atelectasis.

## Materials and Methods

### Ethics Statement

The study protocol was approved by the Ethics Committee of Fukuoka National Hospital (reference number: 24–08), and we obtained written informed consent from all the parents on behalf of the enrolled children. In addition, in cases of elder child, the written consent was obtained from the subjects as well (n = 2).

### Subjects

A total of 15 children with right middle lobe atelectasis and 14 healthy control children participated in this study between August 2012 and August 2013. The inclusion criteria for the atelectasis group were as follows: (1) subjects with right middle lobe atelectasis during the course of bronchial asthma attacks or pneumonia, (2) subjects between 3 and 7 years of age, and (3) subjects for whom RPT had been prescribed. The diagnosis and follow up of right middle lobe atelectasis were performed using chest radiograph in both anteroposterior and lateral views [7]. The chest radiograph was taken every three to four days. All the chest radiographs were reviewed by the pediatricians in charge of the patients. During the course of the disease, subjects were treated according to the standard of care at our hospital including RPT, bronchodilator therapy, antibiotic therapy, and oxygen therapy, if needed. All the subjects received standard RPT 3–4 times daily using a continuous positive airway device (Ez-PAP; Smiths Medical Japan, Ltd.) [8]. Subjects were instructed to breathe 50 times normally through the mask during each session. The same registered physiotherapist administered RPT to all the subjects. The healthy subjects were recruited from children who lived near the hospital and had not exhibited any respiratory symptoms for at least one month.

### Lung sound measurements

The lung sounds were recorded using a custom-made recording system for lung sounds while the subject was in a quiet room in the hospital. The recording system was comprised of two air-coupled microphones (ECM-PC60; SONY, Japan) and an IC recorder (ICR-PS285RM; SANYO, Japan). Microphones were attached to two sites on the chest wall (bilaterally at the fifth intercostal spaces on the mid-clavicular line) with a custom made attachment and double-sided adhesive tape. The lung sounds were recorded by the IC recorder as digital data with a 16-bit resolution and a sampling frequency of 44.1 kHz.

For the subjects with atelectasis, the recording was performed before the start of RPT and after the radiographical resolution of atelectasis. For the control subjects, the lung sounds were only recorded once. The subjects were instructed to breathe slowly and deeply for 30 seconds while in a supine position. The examiner used hand signals to direct the inhalations and exhalations.

### Lung sound analysis

**Fig 1:**
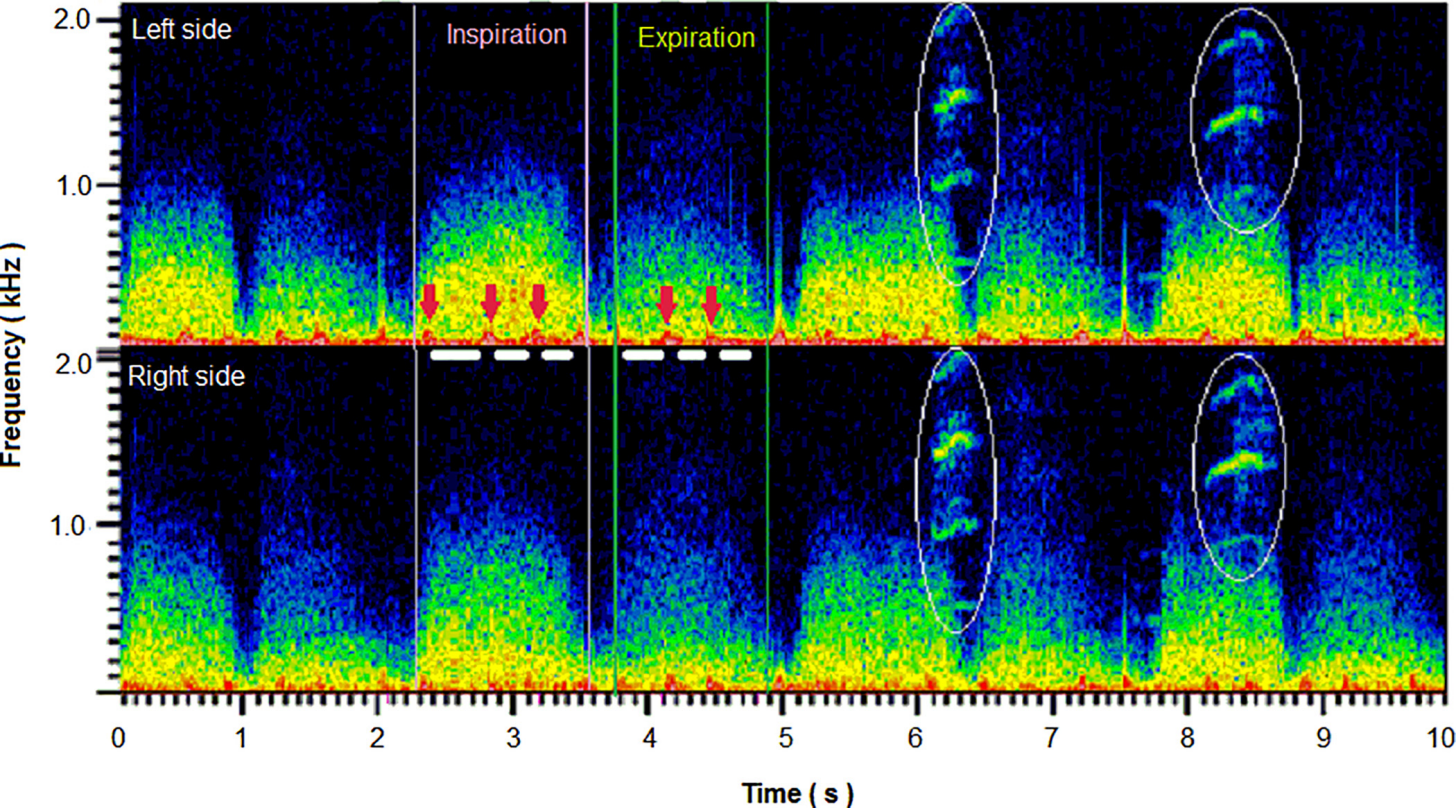
Example of the analytic procedure. Sound spectrographs were displayed on a monitor, and segments suitable for analysis were selected. The octave bands sound pressure levels were calculated as the averaged value for the segments (white horizontal bars) without noise contamination (heart sounds; red arrows). Spectra in the white ellipses indicate contaminated voice sounds of another child during one respiratory phase.

The recorded sounds were analyzed using a custom-made computer program. The program visualized the two lung sound channels as a spectrogram, and appropriate segments were selected for analysis. Thereafter, data segments that included adventitious sounds or various noises were excluded based on a visual inspection of the sound spectrogram. Consequently, an average of 10 breaths were selected for each subject (Fig 1). For each selected segment, the sound pressure level in three octave bands (100–200 Hz, 200–400 Hz, and 400–800 Hz) were calculated using a fast Fourier transform (2,048 points, Hanning window). The recording system was calibrated using a reference sound pressure (94 dB [0 dB = 20 μPa], 1 kHz), and all the data were expressed as dB SPL. In addition, the adventitious sounds were evaluated using a spectrogram and a time-expanded waveform according to the Computerized Respiratory Sound Analysis (CORSA) guidelines [9].

### Statistical analysis

The octave band sound pressure levels (in dB) were averaged separately for inspiration and expiration in each subject. Using these data, the right-to-left ratios (right value [dB]–left value [dB]: R/L ratio; see Appendix) of the octave bands sound pressure were calculated to quantify the difference in right and left breath sound intensities. The expiration-to-inspiration ratios (expiratory value [dB]–inspiratory value [dB]: E/I ratio) of the octave bands sound pressure were also calculated. Comparisons between the two groups and before and after treatment were made using the independent t-test and the paired t-test, respectively. The analyses were performed using EZR (Saitama Medical Center, Jichi Medical University, Saitama, Japan). A value of *P* < .05 was considered statistically significant.

## Results

### Subject characteristic

The subject characteristics are presented in Table 1. Fifteen atelectatic children and 14 healthy children were recruited for this study, but 2 atelectatic children declined to participate. Consequently, 13 children (5 males, 8 females) with atelectasis and 14 healthy children (8 males, 6 females) were registered. Children with atelectasis had various respiratory conditions: bronchial asthma attacks (n = 2), pneumonia (n = 4), and bronchial asthma attacks and pneumonia (n = 7). In children with atelectasis, the mean duration from the start of RPT until radiographical resolution of the atelectasis was 6.5 days (SD, 2.1). This relatively short duration may be related to the early intervention for atelectasis in this group (duration from symptom onset to RPT start: 3.3±1.3 days). Four children were provided with supplemental oxygen at the baseline.

**Table 1:** Subject characteristics.

Characteristics	Controls	Atelectasis subjects
Subjects (n)	14	13
Age (years)	5 .6 (1.5)	5.0 (1.4)
Male/female (n)	8/6	5/8
Diagnoses (n)		
Pneumonia, Asthma/atelectasis		7
Pneumonia/atelectasis		4
Asthma/atelectasis		2
Duration of atelectasis (d)		
From symptom onset to RPT start		3.3 (1.3)
From RPT start to recovery		6.5 (2.1)
Oxygen therapy at the baseline (n)		4

Mean (SD) values are presented for age and days. Definition of abbreviations: RPT = Respiratory physical therapy.

### Baseline breath sound sound pressure levels at the right middle lung field

The mean sound pressure levels of breath sounds at the right middle lung fields at baseline are shown in Table 2. The values were statistically not different between the atelectasis and control groups.

**Table 2:** Baseline breath sound sound pressure levels over the right middle lobe in the control and atelectasis subjects.

Respiratory phase	Frequency band	Sound pressure level (dB)		P value
		Controls	Atelectasis subjects	
Inspiration	100–200Hz	80.1 (4.4)	77.6 (3.8)	0.13
	200–400Hz	78.3 (4.3)	75.8 (4.4)	0.15
	400–800Hz	65.9 (4.9)	64.1 (6.3)	0.42
Expiration	100–200Hz	74.4 (4.7)	72.7 (4.4)	0.33
	200–400Hz	68.4 (5.8)	66.9 (5.4)	0.50
	400–800Hz	54.2 (5.6)	55.3 (5.5)	0.60

Mean (SD) values are presented. Student’s unpaired t-test was used to test the difference between controls and atelectasis subjects.

### R/L ratio

Before treatment, the inspiratory R/L ratios were significantly lower in the atelectasis group than in the control group for all the frequency bands. After the resolution of atelectasis, the inspiratory R/L ratios for all the frequency bands remained significantly lower in the atelectasis group than in the control group. The expiratory R/L ratios for any of the 3 frequency bands were not significantly different between the two groups before treatment, but the ratios for the 200–400 Hz bands became significantly lower in the atelectasis group than in the control group after the resolution of atelectasis (Table 3).

**Table 3:** Comparison of right-to-left ratios (R/L ratios) of breath sound sound pressure between controls and atelectatic subjects.

Respiratory phase	Frequency band	R/L ratio (dB)			P value		
		Controls	Atelectasis subjects		Controls vs. Atelectasis		Atelectasis
			Before treatment	After resolution	Before	After	Before vs. After
Inspiration	100–200Hz	1.0 (2.7)	-2.2 (2.1)	-1.4 (2.2)	0.02	0.02	0.35
	200–400Hz	-1.4 (2.3)	-4.8 (2.5)	-3.8 (2.7)	0.001	0.02	0.27
	400–800Hz	-1.7 (2.7)	-5.9 (2.3)	-5.1 (4.4)	0.007	0.03	0.50
Expiration	100–200Hz	1.5 (3.2)	-0.6 (3.3)	-1.1 (3.2)	0.11	0.06	0.54
	200–400Hz	2.4 (3.2)	-0.1 (3.9)	-1.2 (2.6)	0.08	0.003	0.28
	400–800Hz	0.5 (3.0)	-1.5 (2.3)	-1.6 (3.2)	0.06	0.08	0.94

R/L ratio: right breath sound sound pressure (dB)–left breath sound sound pressure (dB). Mean (SD) values are presented. Student’s unpaired t-test was used to test the difference between controls and atelectasis subjects, and paired t-test was used to test the difference between before and after treatment.

### E/I ratio

Before treatment, the E/I ratios for all the frequency bands were significantly higher in the atelectasis group than in the control group. After the resolution of atelectasis, the E/I ratio decreased for all the frequency bands and became similar to that in the control group (Table 4).

**Table 4:** Comparison of expiration-to-inspiration ratios (E/I ratios) of breath sound sound pressure for the right middle lobe between controls and atelectatic subjects.

Frequency band	E/I ratio (dB)			P value		
	Controls	Atelectasis subjects		Controls vs. Atelectasis		Atelectasis
		Before treatment	After resolution	Before	After	Before vs. After
100–200Hz	-6.6 (2.0)	- 4.8 (2.1)	- 6.5 (3.2)	0.03	0.91	0.01
200–400Hz	-11.9 (2.9)	- 9.0 (3.6)	-12.1 (2.4)	0.02	0.84	0.007
400–800Hz	-13.5 (2.6)	- 9.5 (3.7)	-11.0 (3.7)	0.01	0.06	0.001

E/I ratio: expiratory breath sound sound pressure (dB)–inspiratory breath sound sound pressure (dB). Mean (SD) values are presented. Student’s unpaired t-test was used to test the difference between controls and atelectasis subjects, and paired t-test was used to test the difference between before and after treatment.

### Adventitious sounds

**Fig 2:**
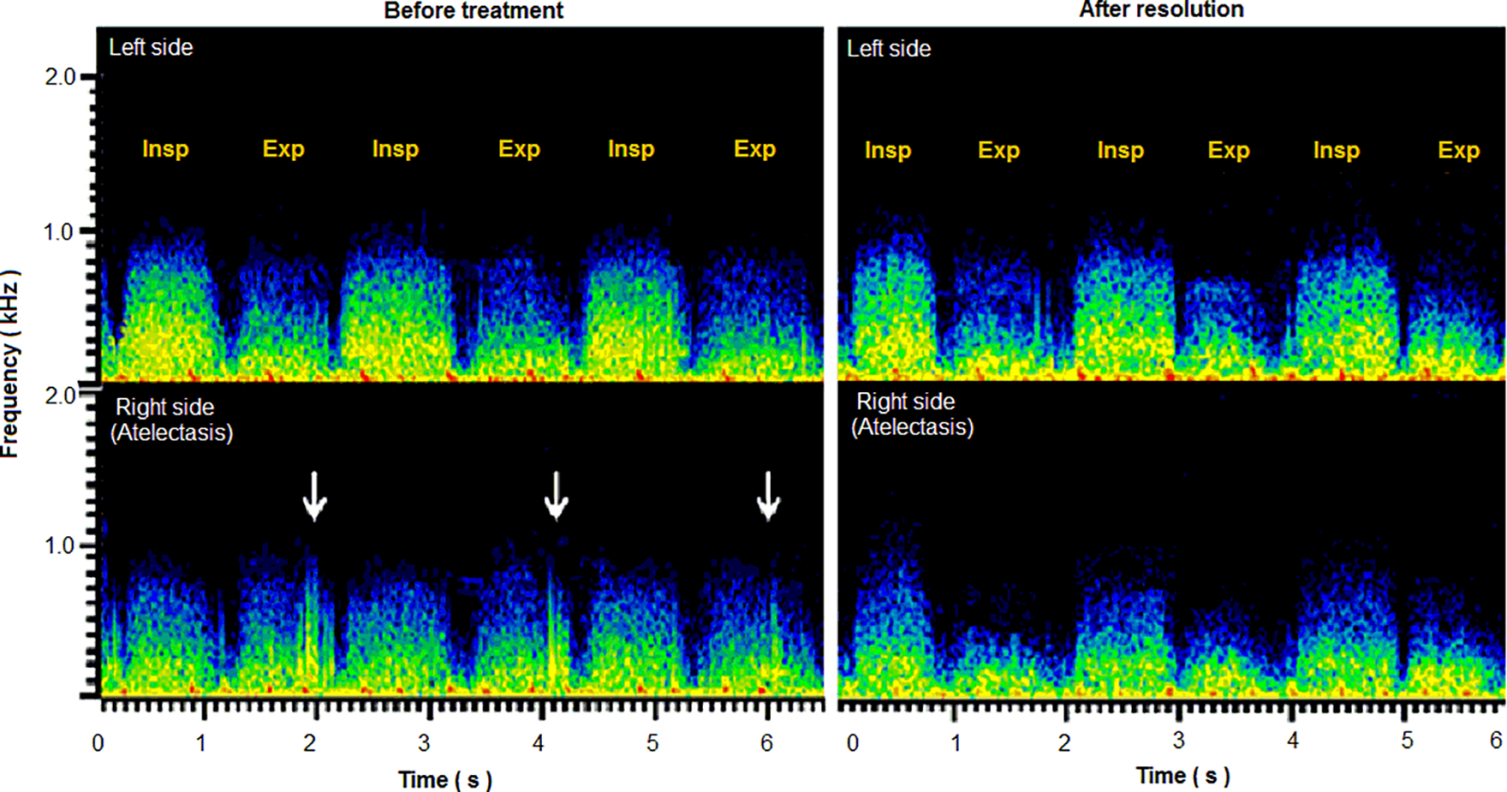
Representative sound spectrograms in a child with right middle lobe atelectasis before and after treatment. The sound spectrograms for recordings over bilateral middle lung fields are shown. Before treatment, the inspiratory sound intensity was lower on the right than on the left. Furthermore, on the right (affected) side, the expiratory sound intensity was similar to the inspiratory sound intensity. In addition, coarse crackles (white arrows) were identified. After resolution, the right-to-left difference in inspiratory sound intensity persisted. On the other hand, the recovery of a normal pattern of inspiratory breath sound dominance over expiratory breath sound was observed and the adventitious sounds disappeared.

Before treatment, adventitious sounds were heard in 7 subjects with atelectasis (coarse crackles, n = 4; rhonchi, n = 3). After the resolution of atelectasis, these sounds disappeared in all the subjects. A typical sound spectrogram of a subject with adventitious sounds is presented in Fig 2.

## Discussion

Using computerized acoustic measurements, we analyzed the lung sounds of children with right middle lobe atelectasis and compared them with those of healthy children to clarify the characteristics of the breath sounds and their changes after the resolution of atelectasis. Our study had two major findings. First, before treatment, the inspiratory R/L ratios for all the frequency bands were significantly lower and the E/I ratios for all the frequency bands were significantly higher in the atelectasis group than in the control group. Second, after the resolution of atelectasis, the inspiratory R/L ratios remained unchanged, while the E/I ratios decreased for all the frequency bands and became similar to those in the control group.

We calculated the R/L ratio to estimate the difference between right and left lung sound intensities, which can be perceived by chest auscultation. As anticipated, before treatment, the inspiratory R/L ratios were lower in the atelectasis group than in the control group for all the frequency bands. However, after the resolution of atelectasis, the R/L ratios remained lower in the atelectasis group than in the control group in all the frequency band. We examine the change in lung sound separately for the three octave bands. Lung sound powers are known to have different origins depending on the frequency band. Although the dominant breath sound spectra on the chest wall exist at below 200 Hz, this frequency band is highly affected by musculoskeletal noises generated from thorax movements and muscle contractions during respiration [10]. Pasterkamp et al. demonstrated that turbulent airflow in the respiratory tract is the major determinant of lung sound power only at frequencies above 300 Hz, suggesting that breath sound powers above 300 Hz reflect local ventilation [11]. Therefore, the persistence of decreased inspiratory sound powers above 200 Hz may reflect the persistence of airflow restrictions even after the radiographical resolution of atelectasis. However, a long-term follow-up study is needed to confirm this phenomenon. Moreover, it should be also considered that radiological resolution may not always mean complete recovery from atelectasis. In addition, a slight but significant right-to-left lung sound intensity difference was found in the control group. This finding is not unusual because such laterality of lung sounds has been reported as a normal phenomenon because of physiological differences in local ventilation and transmission properties [12].

During chest auscultation, the judgment of “normal” or “abnormal” breath sounds is based on the difference between inspiratory and expiratory breath sound intensities as well as the laterality of breath sounds in terms of intensity and pitch. In the present study, the E/I ratio was used to estimate the tendency for bronchial breathing (abnormal breath sounds). The E/I ratios for all the frequency bands were significantly higher in the atelectasis group than in the control group before treatment, but the ratios became similar to those in the control group after the resolution of atelectasis. This change indicated that the bronchial sounds returned to normal breath sounds (vesicular sounds). Furthermore, this change may reflect the normalization of the sound transmission property of the lung [13], which had been heightened because of the atelectasis.

Sound transmission abnormalities of the lung are also known to occur in pneumonia. Shirota and colleagues studied the lung sounds of consolidated lungs caused by pneumonia in children and reported decreased inspiratory and increased expiratory sound powers, resulting in a higher E/I ratio, with normalization after the resolution of pneumonia [14]. Their results showed a more definite change in bronchial breathing sounds than the results of the present study. In our cases, E/I ratio was not affected by the cause of atelectasis (pneumonia or bronchial asthma; data not shown). Therefore, the difference was probably caused by differences in the sound transmission properties between volume-preserved consolidation (pneumonia) and volume-reduced atelectasis.

Another important auscultation finding in the course of atelectasis was change in adventitious sound. Before treatment, adventitious sounds were heard in 7 subjects, and after the resolution of atelectasis, these sounds disappeared in all the subjects. The main cause of adventitious sounds in our cases (coarse crackles, rhonchi) is supposed to be retention of airway secretions. The disappearance of adventitious sounds may reflect the effect of treatments including pharmacological and physiological therapies.

The present study has a few limitations. First, we did not measure the airflow, which is usually required because the lung sound sound pressure is proportional to the airflow squared [15–17]. This issue is considered one of the reasons why baseline breath sound sound pressure levels were not different between the atelectasis and control groups. However, the measurement of airflow is not always easy in children. Instead, we measured the lung sounds on both sides simultaneously during deep breathing and calculated the right-to-left ratio of the lung sound sound pressure. We thought that this method was suitable for estimating changes in the lung sounds as perceived by chest auscultation. Second, the lung sounds were recorded in a quiet but not soundproof room in the hospital. Therefore, the measured lung sounds might have been contaminated by environmental noises. However, the influence of environmental noise was minimized by excluding noise-contaminated segments by visual inspection of the spectrogram. Third, the number of cases of this study was too small to have enough statistical power in some comparisons. If there were much number of cases, the difference between before and after treatment R/L ratio may be statistically significant. However, the difference was trivial (≦1dB) and may be clinically not relevant.

The present study has important clinical implications. Regarding auditory discrimination, a study by Jesteadt and colleagues has shown that a relative discriminatory intensity threshold is equivalent to a difference of approximately 1–1.5 dB [18,19]. In this regard, after the resolution of atelectasis, the mean E/I ratio in the atelectasis group decreased significantly and by more than the relative discriminatory sound intensity threshold in all the frequency bands. On the other hand, the change in the R/L ratio before and after the resolution of atelectasis was not significant and did not meet the relative discriminatory threshold for sound intensity. Consequently, the difference between inspiratory and expiration sound intensities (in other words, the recovery of vesicular sounds) might be an important auscultation sign for evaluating improvements in atelectasis. We speculate that remained low inspiratory R/L ratio reflects persistent decrease in local ventilation while normalized E/I ratio reflects re-expansion of lung parenchyma.

In conclusion, the objective measurement of lung sounds suggested that the normalization of a breath sound characteristic, i.e., the inspiratory-to-expiratory intensity difference, might be a more important marker of atelectatic improvement than the right-to-left breath sound intensity difference in children.

## Appendix

We designated the difference between the right (Rt) and left (Lt) sound pressure level in dB as right-to-left ratio (R/L ratio) because dB is a logarithmic scale.

$$\text{Rt}\;(\text{dB})=20{\times log}_{10}\left(\frac{Prt}{P_{0}}\right)$$

$$Lt\;(dB)=20\times {log}_{10}\left(\frac{Plt}{P_{0}}\right)$$

$$Rt-Lt=20\times \left({log}_{10}(\frac{Prt}{P_{0}})-{log}_{10}(\frac{Plt}{P_{0}})\right)=20\times \left({log}_{10}(Prt)-{log}_{10}(Plt)\right)=20\times {log}_{10}\left(\frac{Prt}{Plt}\right)$$

Prt, Plt: the right and left sound pressure (root mean square value of sound pressure)

    P_0_: the reference sound pressure

Thus, the difference in dB means the ratio expressed as dB.

It is well known that the human ear does not perceive an absolute difference, but rather a ratio between two values. Therefore, the R/L and E/I ratios are considered to be reasonable variables.
